# Cataracta Pulverulenta in Marfan Syndrome: An Atypical Ocular Presentation: Case Report

**DOI:** 10.22336/rjo.2025.90

**Published:** 2025

**Authors:** Chhaparia Geetanjali, Kumar Santosh, Singh Vinod Kumar, Singh Manendra, Rawat Ayusha, Verma Sonam, Beg Mirza Maariyam

**Affiliations:** 1 Regional Institute of Ophthalmology, Prayagraj, Uttar Pradesh, India

**Keywords:** Marfan syndrome, nucleus pulverulenta, lens subluxation, pectus excavatum, cataract, fibrillin, BE = Both eyes, RE = Right eye, LE = Left eye, IOL = Intraocular Lens, MSICS = Manual Small Incision Cataract Surgery, OPD = Outpatient Department, ASD = Atrial Septal Defect, BSS = Balanced Salt Solution, FBN = Fibrillin, DNA = Deoxyribonucleic Acid

## Abstract

**Aim:**

To report a rare ocular presentation of cataracta pulverulenta in a patient with Marfan syndrome in the absence of classical lens subluxation.

**Method:**

A 14-year-old male presenting with severe bilateral diminution of vision underwent ophthalmic examination and systemic workup. Slit-lamp biomicroscopy, echocardiography, and physical assessment were performed. The patient underwent manual small incision cataract surgery (MSICS) with intraocular lens implantation.

**Result:**

Slit-lamp evaluation revealed bilateral cataracta pulverulenta without ectopia lentis. Systemic findings included long arm span (arm span to height ratio of 1.35), high-arched palate, pectus excavatum, nasal voice, and echocardiographic evidence of a 9 mm atrial septal defect with trace mitral and tricuspid regurgitation. The systemic Ghent score exceeded the diagnostic threshold for Marfan syndrome. Postoperative corrected visual acuity improved to 6/6 in the operated eye.

**Discussion:**

Marfan syndrome is traditionally associated with ectopia lentis as its most characteristic ocular manifestation; however, this case demonstrates that cataracta pulverulenta may occur as an uncommon and diagnostically significant presentation even in the absence of lens displacement. The detection of fine, central nuclear lens opacities in a pediatric patient should raise clinical suspicion of an underlying connective tissue disorder, particularly when subtle skeletal and cardiovascular features coexist. Abnormal fibrillin-1 may contribute to atypical lens development, accounting for such non-classical cataract patterns in Marfan syndrome. Recognition of this rare presentation is essential, as it facilitates early systemic evaluation and confirmation using the Ghent criteria, allowing timely multidisciplinary care. Moreover, the excellent visual recovery following manual small incision cataract surgery highlights that early surgical management of syndromic cataracts can yield favorable outcomes and underscores the essential role of ophthalmologists in identifying systemic diseases through ocular signs.

**Conclusion:**

While ectopia lentis is the most common ocular manifestation of Marfan syndrome, cataracta pulverulenta may also be a rare but significant finding. This case underscores the importance of thorough ocular and systemic evaluation in pediatric cataract patients, as ophthalmic anomalies may be the first clue to underlying systemic connective tissue disorders, such as Marfan syndrome.

## Introduction

Nucleus pulverulenta refers to a type of congenital cataract marked by a fine, dust-like or powdery clouding of the lens, specifically affecting the embryonic and fetal nuclei [**1**].

Marfan syndrome is an inherited connective tissue disorder passed down in an autosomal dominant pattern. It results from mutations in the *FBN1* gene, which codes for the protein fibrillin-1. This gene is found on chromosome 15 at location q21. A mutation in one of the two *FBN1* gene copies leads to abnormal fibrillin-1 production, compromising the structure and function of connective tissue throughout the body [**2**].

## Case report

**Fig. 1:**
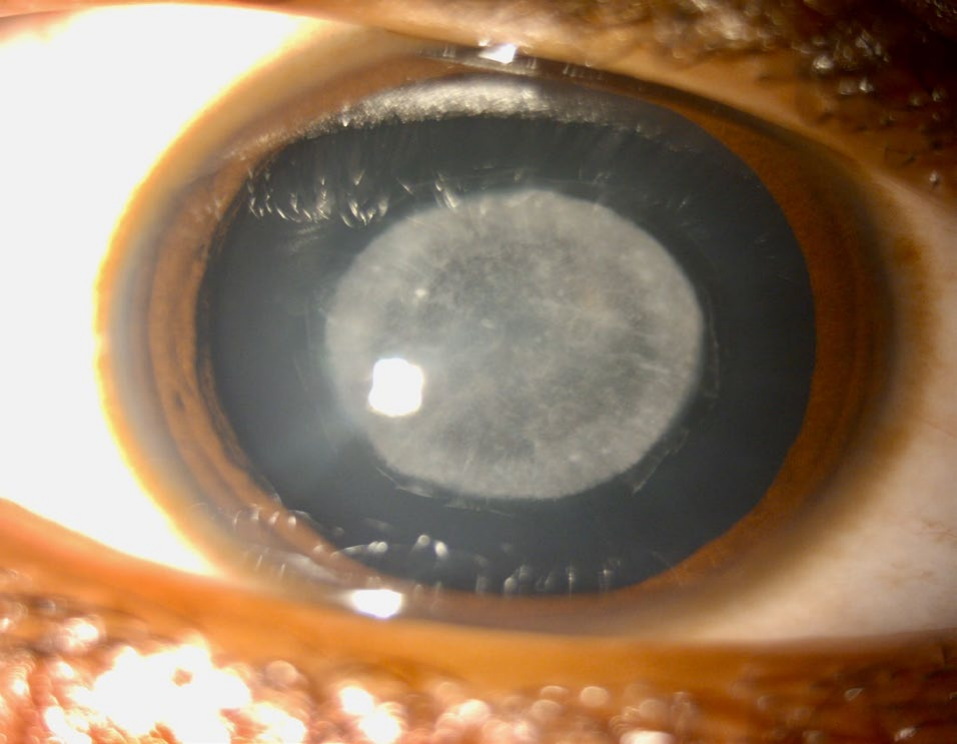
Slit-lamp image showing cataracta pulverulenta under diffuse illumination, characterized by a dust-like opacity in the central lens nucleus

**Fig. 2:**
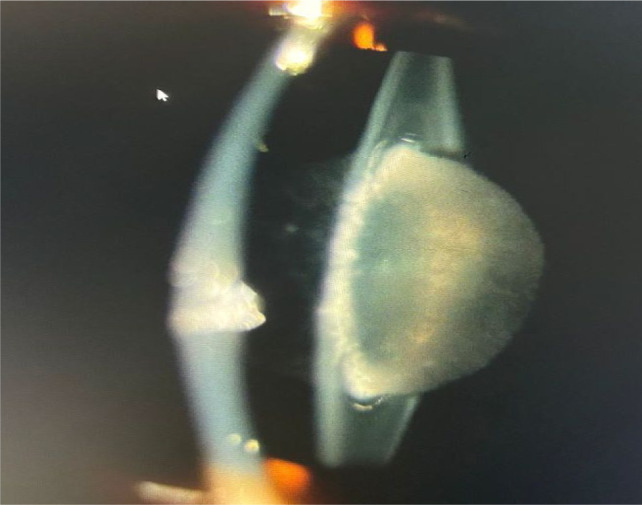
Slit-lamp image of the same eye under slit section view, clearly outlining the central nuclear opacities typical of cataracta pulverulenta

A 14-year-old male presented to the ophthalmology outpatient department with complaints of marked visual impairment, particularly under bright light conditions. Slit-lamp examination revealed a clear anterior chamber; however, the lens nucleus appeared cataractous (**Fig. 1**). Based on the characteristic powdery opacities observed in the lens, a diagnosis of cataracta pulverulenta was established (**Fig. 2**) [**3**].

**Fig. 3:**
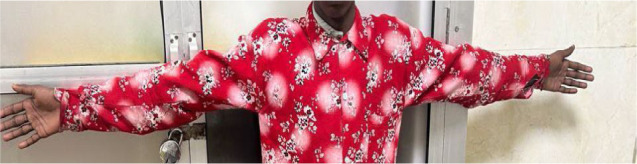
Clinical photograph demonstrating long arm span, with an arm span to height ratio of 1.35-supportive of Marfan syndrome

**Fig. 4:**
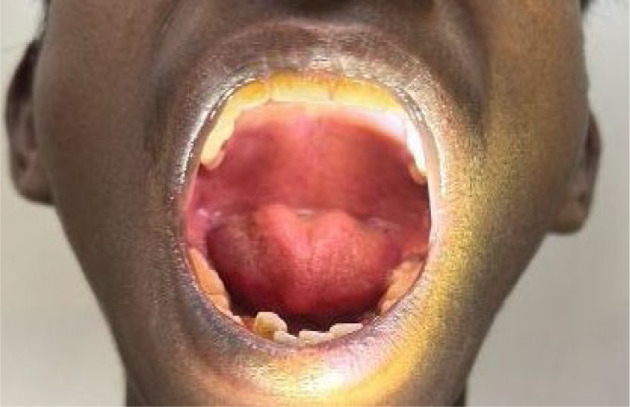
Intraoral view showing a high-arched palate, a common craniofacial feature observed in Marfan syndrome

**Fig. 5:**
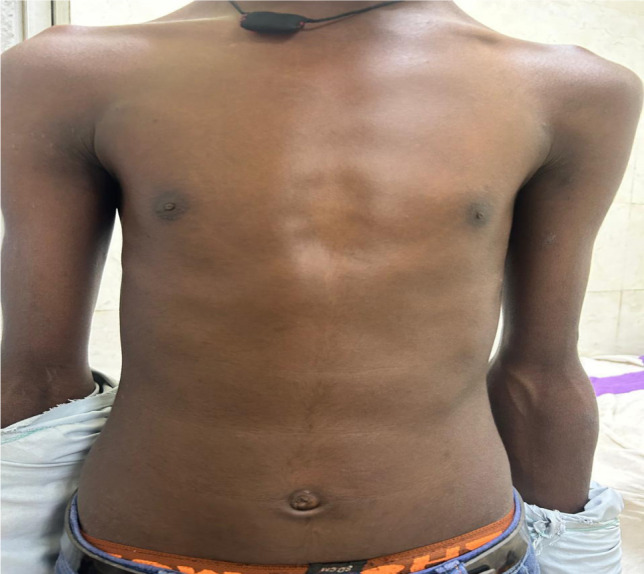
Image depicting pectus excavatum - a sunken chest wall deformity often associated with connective tissue disorders like Marfan syndrome

Systemic examination revealed several phenotypic features suggestive of a connective tissue disorder, including a high-arched palate with a nasal voice, increased arm span with arachnodactyly, and an arm span-to-height ratio of 1.35 (**Fig. 3-5**). Additional skeletal abnormalities included pectus excavatum. Echocardiographic assessment demonstrated trace tricuspid and mitral valve regurgitation, along with a small atrial septal defect measuring 9 mm.

The patient met the revised Ghent criteria with a systemic score exceeding 7, confirming the diagnosis of Marfan syndrome. Although the classical ocular manifestation of ectopia lentis (lens subluxation) was absent, the presence of cataracta pulverulenta as the primary ophthalmic finding prompted further evaluation for Marfan syndrome. We reported this case to highlight the atypical presentation of cataracta pulverulenta in a patient with Marfan syndrome in the absence of lens subluxation—a rare and noteworthy observation [**4**].

## Discussion

**Fig. 6:**
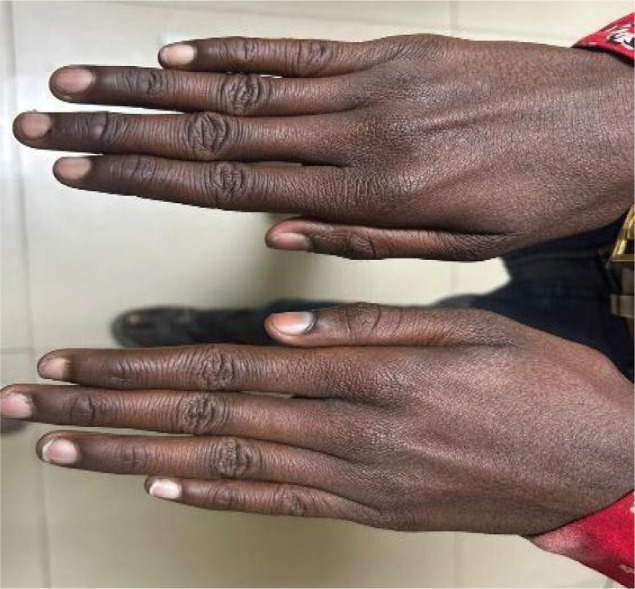
Photograph showing arachnodactyly (long, slender fingers), another hallmark skeletal feature in Marfan syndrome

The patient was taken up for manual small incision cataract surgery (MSICS) under peribulbar anesthesia following full pharmacologic pupil dilation. A superior rectus bridle suture was placed to facilitate globe stabilization. A fornix-based conjunctival flap was fashioned, and hemostasis was achieved using bipolar diathermy. A frown-shaped partial-thickness scleral incision was made approximately 3 mm posterior to the limbus using a crescent blade, with a depth of about 0.3 mm. Dissection was carefully advanced laterally to create the scleral tunnel (**Fig. 6**) [**5**].

Upon reaching the limbus, the tunnel was extended into clear cornea using anterior-posterior movements of the blade, transforming the incision into a lamellar flap. Scleral side pockets were created on either side of the tunnel to enhance wound stability. The anterior lens capsule was stained with trypan blue, and the dye was instilled beneath an air bubble to minimize exposure of the corneal endothelium. A viscoelastic agent was injected to deepen the anterior chamber and provide intraocular protection [**6**].

A continuous curvilinear capsulorhexis (CCC) of approximately 5 mm diameter was performed using a cystotome. A keratome was then introduced through the tunnel, angled downward, and advanced to enter the anterior chamber, thereby completing the internal corneal incision. Multiple hydrodissections were performed to loosen the cortical–nuclear complex and allow controlled prolapse of the nucleus into the anterior chamber. The nucleus was then engaged with an irrigating lens loop and extracted through the tunnel, while gentle posterior pressure was applied to the tunnel’s inner lip to facilitate smooth delivery [**7**].

**Fig. 7:**
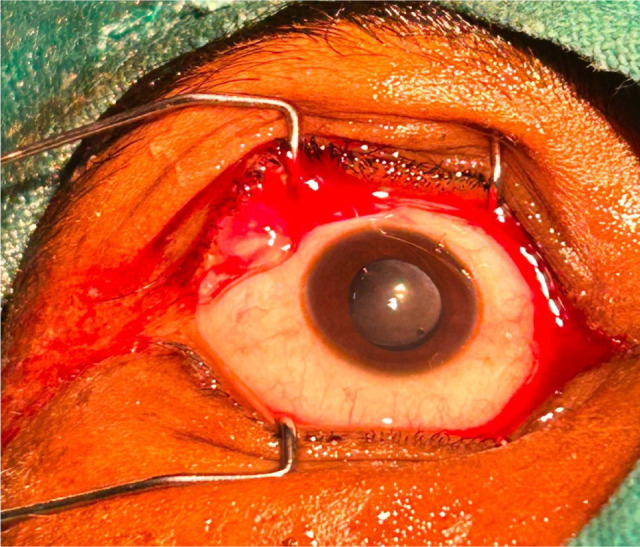
Postoperative image showing the eye after successful manual small incision cataract surgery (MSICS), with a clear cornea and a stable intraocular lens

**Fig. 8:**
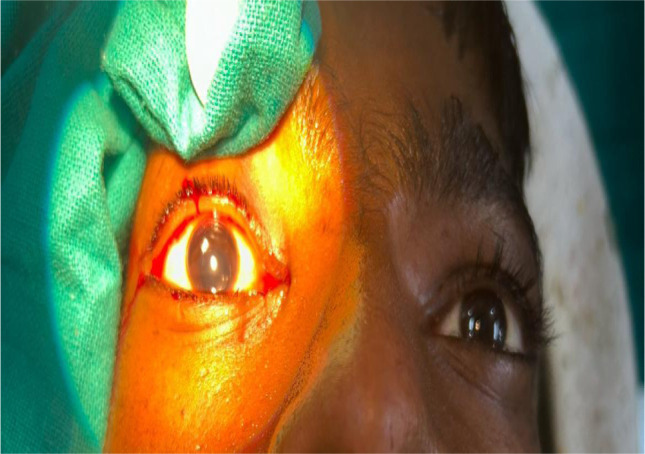
Another postoperative view confirming a successful surgical outcome with a well-centered intraocular lens and quiet anterior chamber

Residual cortical matter was aspirated using a 23-gauge Simcoe cannula. The anterior chamber was thoroughly irrigated with Balanced Salt Solution (BSS) and reformed with viscoelastic. A posterior chamber intraocular lens (PCIOL) was implanted in the capsular bag and positioned using a lens manipulator. The remaining viscoelastic was evacuated using the Simcoe cannula. The paracentesis port was sealed by stromal hydration using BSS injected at the wound margins. Subconjunctival gentamicin and dexamethasone were administered, and the conjunctival flap was secured with bipolar cautery (**Fig. 7, 8**) [**8**].

Postoperative best-corrected visual acuity (BCVA) was recorded at 6/6.

This case report highlights a rare ocular presentation in a patient with Marfan syndrome. Although ectopia lentis is a classical diagnostic criterion, this patient exhibited cataracta pulverulenta in the absence of lens subluxation. Given the central role of ocular signs in raising suspicion for Marfan syndrome, this unusual manifestation is clinically significant. It underscores the importance of comprehensive ophthalmic evaluation in identifying systemic connective tissue disorders and reinforces the critical role ophthalmologists play in their early diagnosis and multidisciplinary management [**9**].

## Conclusion

This case highlights cataracta pulverulenta as a rare and atypical ocular manifestation of Marfan syndrome occurring in the absence of ectopia lentis. It emphasizes the need for a high index of suspicion and comprehensive systemic evaluation in pediatric patients presenting with congenital or developmental cataracts. Early identification of such uncommon presentations can facilitate timely diagnosis of underlying connective tissue disorders and enable appropriate multidisciplinary management. Favorable visual outcomes following surgical intervention further support the role of prompt cataract surgery in improving vision and quality of life in these patients.
